# Socioeconomic disparities in child malnutrition: trends, determinants, and policy implications from the Kenya demographic and health survey (2014 - 2022)

**DOI:** 10.1186/s12889-024-21037-z

**Published:** 2025-01-24

**Authors:** Amos O. Okutse, Henry Athiany

**Affiliations:** 1 Department of Biostatistics, Brown University School of Public Health, Providence, RI USA; 2 Department of Statistics and Actuarial Sciences, School of Physical and Mathematical Sciences, Jomo Kenyatta University of Agriculture and Technology, Nairobi, Kenya; 3 Digital Health Applied Research Center, Jomo Kenyatta University of Agriculture and Technology, Nairobi, Kenya

**Keywords:** Child malnutrition, Decomposition, Socioeconomic inequality, Kenya, Stunting, Underweight, Wasting, Demographic health survey

## Abstract

**Background:**

Child malnutrition remains a critical public health problem, with socioeconomic factors playing a significant role. Socioeconomic factors include household income, parental education, and access to healthcare, which influence a child’s nutritional status. Despite overall progress in reducing under-five child malnutrition in Kenya, disparities persist. This paper analyzes changes, and determinants of child malnutrition, contributions of these determinants to health inequality, and their potential use in the screening for chronic malnutrition in children under five years.

**Methods:**

We use data from the Kenyan Demographic and Health Survey (KDHS 2014 and 2022) and analyze malnutrition using three indicators: Stunting, underweight, and wasting. The determinants of malnutrition are analyzed using multivariate logistic regression. Trends in socioeconomic inequality are analyzed using concentration indices and visualized using concentration curves. Wagstaff decomposition is used to explore the contributions of determinants to inequality in child malnutrition. We investigate diagnostic utility using sensitivity, specificity, predictive values, and area under the ROC curve.

**Results:**

Socioeconomic inequality in under-five child malnutrition increased between 2014 and 2022, with children from the poorest socioeconomic quintiles being disproportionately affected. A child’s age (in months) (Adjusted Odds Ratio [AOR] = 1.01; 95% Confidence Interval [CI]: 1.01 – 1.02), being born to a household in the poorest socioeconomic quintile (AOR = 2.67; 95%CI: 1.92 - 3.72), and sex (male) (AOR = 1.50; 95%CI: 1.35 – 1.67) were associated with an increased risk of stunting. The mother’s age, sex of the child (male), and household socioeconomic status (poorest) was associated with an increased risk of being underweight and wasted, whereas residence was associated with an increased risk of wasting alone after adjusting for potential confounders. A household’s socioeconomic status was the largest contributing factor to health inequality. Sensitivity, specificity, and AUC values were 67.4% (95% CI: 66.4% – 68.4%), 50.6% (95%CI: 50.0% - 51.1%), and 0.59 (95%CI: 0.58 – 0.60), respectively, when using socioeconomic status as a screening tool for stunting.

**Conclusion:**

Socioeconomic disparities are a major barrier to reducing child malnutrition in Kenya, with children from lower socioeconomic quintiles at a greater risk of stunting, underweight, or wasting. This study identifies a child’s sex, age, and household socioeconomic status as key predictors of malnutrition, highlighting the need to include these factors in public health interventions. Addressing these disparities with targeted strategies considering immediate health risks and underlying socioeconomic challenges is essential for equitably improving child health outcomes.

## Introduction

Child malnutrition remains a dominant public health problem globally. While there has been some progress towards attaining the global nutrition target to eliminate all forms of malnutrition by 2030 as part of the Sustainable Development Goals (SDGs), this progress has been slow, and malnutrition levels persist. In 2022, about 148.1 million (22.3%) children below five years were stunted, whereas 45 million (6.8%) and 37 million (5.6%) were wasted and overweight, respectively. Asia and Africa account for 52% and 43% of the global share of children affected by stunting [1]. Moreover, current evidence suggests that the global target of reducing the number of stunted children by 2030 will be missed by more than 39.5 million children, with over 80% of these children expected to be living in Africa [1]. The double and triple burdens of malnutrition, which refer to the coexistence of undernutrition, overnutrition, and micronutrient deficiencies, highlight the complexity of this condition and ultimately contribute to the high susceptibility of children under five years. According to nutrition statistics, 3.62% of all children under the age of five years (15.95 million) have been reported to be both stunted and wasted, whereas 1.87% of all children (8.23 million) have been reported to experience both stunting and overweight globally [2].

Malnutrition is “a state of nutrition in which a deficiency, or an excess, of energy, protein, and micronutrient causes measurable adverse effects on tissue/body form (body shape, size, and composition), function, and clinical outcome” [3]. In children, malnutrition is characterized by three indicators: stunting, underweight, and wasting. Stunting refers to low height for age (too short for age) and reflects growth in linear terms achieved at the age at which the measurements were taken. Conversely, underweight denotes low weight for age (too thin for age) resulting from a short-term lack of food. In contrast, wasting is severe undernutrition (too thin for height) resulting from inadequate food intake and infections [4]. In children under five years, stunting is the preferred measure of overall health and well-being capable of highlighting salient social disparities [5]. Moreover, because stunting measures linear growth in children, it is considered an accurate measure of malnutrition in the long term due to its insensitivity to variations in food consumption [6, 7].

Child malnutrition has been attributed to several diverse interlinked factors. These factors have ranged from child sex [8], birth order [8], age [9], maternal education [8–10], public service utilization [8], and household socioeconomic status [8, 9, 11], and have detrimental short and long-term effects [12, 13]. Not only does child malnutrition affect the physical and cognitive development of a child, it also drastically increases their risk of infections and contributes negatively to their mortality and morbidity [4, 10, 14–18].

In Eastern Africa, the burden of child malnutrition remains high, with stunting prevalence (32.6%) being significantly higher than the global average (22.0%). Wasting (5.2%) and overweight (4.0%), on the other hand, have a lower prevalence compared to the worldwide average [19]. According to the 2022 Kenya Demographic and Health Survey (KDHS) [20], 18% of children under five are stunted (chronically undernourished), 5% are wasted (acutely malnourished), whereas 3% and 10% are overweight and underweight, respectively. While the overall Kenyan burden of child malnutrition has decreased, undernutrition is estimated to cost the country over US$38.3 billion in Gross Domestic Product (GDP) following workforce labor and productivity losses between 2010 and 2030 [21].

Kenya is classified as a middle-income country based on its Gross National Income (GNI) per capita. Achieving middle-income status indicates progress from such activities as heightened investments across all government sectors and improved productivity. Economic growth is expected to enhance the well-being of a country’s population by, for instance, creating employment opportunities, which translate into increased disposable income, improved health, and education [22, 23]. Improved living standards following economic growth are expected to translate into improved nutritional outcomes for children and adults [24, 25]. However, economic advancement does not guarantee an equitable distribution of benefits across the population; often, these tend to be skewed, with some groups benefiting more than others [4].

Kenya has made notable progress in addressing malnutrition through various initiatives. These include the Baby Friendly Community Initiative [26], which establishes community groups to monitor child growth and provide feeding counseling; the Nutrition and Health Program Plus (NHplus), funded by USAID, which aims to improve national nutrition security; and school feeding programs designed to promote school enrollment, attendance, and concentration by providing children with balanced meals at subsidized prices [27]. However, programs such as the school feeding programs face challenges due to insufficient government funding, resulting in a limited variety of meals that lack essential nutrients crucial for optimal child growth and development. Given the severe threat that malnutrition poses to children’s growth, survival, and overall well-being, it remains a significant concern for the government, public health professionals, and policymakers.

This study aimed to address gaps in knowledge regarding socioeconomic disparities in the Kenyan child malnutrition burden. First, we analyze trends in stunting, underweight, and wasting across socioeconomic groups, geography, and selected household, child, maternal, and paternal characteristics. Second, we explore determinants of child malnutrition and employ standard procedures of inequality analysis to quantify their contributions to health inequality. Finally, we examine the independent clinical utility of household socioeconomic status and significant child characteristics in acute malnutrition screening, and present policy implications of the findings.

## Methods

### Data source

We used data from the 2014 and 2022 Kenya Demographic and Health Surveys (KDHS) (standard DHS survey data) [28, 29]. These surveys adopt a two-stage stratified cluster sampling approach, with clusters sampled in the first stage and households in the second. In 2014, a response rate of 99% was achieved from 39,679 households, and in 2022, a 98% response rate was achieved from 38,731 occupied households [20, 30]. Our analyses considered all live children (0–59 months) of interviewed mothers, excluding those with missing anthropometric data, including height-for-age, weight-for-age, and weight-for-height. The data was weighted for non-response and used with DHS authorization.

### Variables

#### Outcome

Malnutrition was characterized by stunting (low height-for-age z-scores, HAZ), underweight (low weight-for-age z-scores, WAZ), and wasting (low weight-for-height z-scores, WHZ) [4, 11]. In children under five, HAZ, WAZ, or WHZ between −2 and −3 standard deviations (SD) below the median suggests moderate stunting, underweight, or wasting, respectively. Z-scores less than −3 SD below the World Health Organization’s (WHO) child growth standards median indicate severe conditions [31]. We categorized children with HAZ, WAZ, and WHZ scores below −2 SD of the WHO growth standards median as stunted, underweight, and wasted, respectively.

#### Covariates

We considered a comprehensive set of determinants linked to child malnutrition. Child-specific variables included age (in months), sex, place and region of residence, child delivery location, and birth order. At the household level, we accounted for religion and the wealth index as a proxy for socioeconomic status. Maternal indicators were age, education level, and birth interval, whereas the father’s education was considered as a paternal characteristic. Our choice of these covariates is grounded in existing literature and availability in our data set [4, 11, 32].

### Statistical analysis

#### Weighted prevalence of child malnutrition

The weighted prevalence of stunting, underweight, and wasting was estimated in relation to maternal, child, and household characteristics. The data was weighted to represent the population and account for non-response. Overall differences across categories were examined using a design-based Pearson chi-squared test, whereas the significance of differences in group means was analyzed using two-sample t-tests for continuous variables. The significance of the differences in child malnutrition by socioeconomic status between 2014 and 2022 was analyzed using two-sample proportion tests.

#### Disparities in child malnutrition

The extent and trends of socioeconomic disparities in stunting, underweight, and wasting were quantified using concentration indices (CIs) estimated based on the corresponding z-scores [33–35]. Concentration indices quantify socioeconomic disparities in a health variable and allow assessment of the extent and levels of disparities. CIs were computed as double the area between the concentration curve and the line of equality – the $45^{\circ }$ line. According to O’Donnell et al. [33], this is:

1 $$\begin{aligned} CI = \frac{2}{\mu } \text {cov}(h, r) \end{aligned}$$

In Equation (1), $\mu$ is the average of malnutrition (stunting, underweight, and wasting) in children under five, *h* denotes observation-specific child malnutrition, and *r* is the rank of the socioeconomic status of a household. The CI of a given health variable takes values between −1 and +1, with 0 suggesting perfect equity of the health variable between the poorest and the richest socioeconomic groups. Negative values suggest a higher concentration of malnutrition among the poorest socioeconomic group, whereas positive values suggest a higher concentration of inequity among the richest socioeconomic group [4, 9, 11, 35].

#### Determinants of child malnutrition and utility in screening for child stunting

Determinants of child malnutrition were investigated using binary logistic regression. Separate models were fitted for stunting, underweight, and wasting. Odds ratios (OR) were computed for each adjustment covariate to examine associations between malnutrition indicators and the covariates. We used results from the fitted adjusted logistic regression model to inform covariates evaluated for clinical utility in chronic child malnutrition screening, where we focused on significant determinants of child stunting based on this model. Our analyses here focused on child stunting since it suggests chronic malnutrition [4].

We estimated the diagnostic performance of a household’s socioeconomic status and child characteristics, which we found to impact under-five child stunting significantly (i.e., a child’s age and sex). These factors were each used to independently predict a child’s nutritional status (stunting) and compute sensitivity, specificity, predictive values, and area under the receiver operating characteristic curve (AUC) using the  command in Stata [36]. Stunting had a 22.7% (95 %CI: 22.3% - 23.1%) prevalence in this dataset, suggesting a classification problem with imbalanced data since only a tiny proportion of children are stunted. Evaluating the clinical utility of these risk factors would result in highly optimistic results characterized by a high hit ratio but poor explanatory capabilities. We used threshold-moving, a technique for training a cost-sensitive classifier to address the class imbalance. We adjusted the decision threshold to accurately predict the minority class and address class imbalance by setting the optimal threshold based on a random search, maximizing the AUC [37]. The choice of the AUC as a performance metric was informed by its insensitivity to changes in class distribution. An AUC of 0.5 suggests the limited discriminatory ability of a test that is no better than random guessing. Values between 0.7 to 0.8, 0.8 to 0.9 are acceptable, whereas those above 0.9 are considered exceptional [38, 39].

#### Decomposition of socioeconomic inequities in child malnutrition

Contributions of determinants of malnutrition in children under five to the observed socioeconomic disparities were examined through a decomposition analysis. This decomposition was restricted to stunting and underweight, indicators that exhibited substantial differences between 2014 and 2022.

We considered a linear regression model where malnutrition – the response variable (*y*)– was modeled as a linear combination of the *k* determinants ($X_k$) as:

2 $$\begin{aligned} y = \alpha + \sum _{k} \beta _k X_k + \epsilon \end{aligned}$$

where $\alpha$ is the intercept, $\beta _k$ denotes the coefficient of $X_k$ and $\epsilon$ is the error term.

In terms of the CI for the response *y*, Equation (2) becomes:

3 $$\begin{aligned} CI = \sum _{k} \left( \frac{\beta _k \bar{X}_k}{\mu } \right) CI_k + \frac{G CI_{\epsilon }}{\mu } \end{aligned}$$

where $\mu$ denotes the average of *y*, $\bar{X}_k$ denotes the mean of the $k^{th}$ variable, $\beta _k$ denotes the coefficient of each determinant, $CI_k$ denotes the CI of each of the regressors in the model, and $GCI_\epsilon$ denotes the generalized concentration index for the error term, $\epsilon$.

Equation (3) has two components: the explained component $\left( (\beta _k \bar{X}_k)/ \mu )CI_k\right)$ and the unexplained component ($GCI_{\epsilon }/\mu$). In this case, $(\beta _k \bar{X}_k)/\mu$ is the elasticity denoting the effect of each $CI_k$ on the overall CI of the outcome variable, *y*. We employed the Wagstaff normalization technique for the CI values given our use of binary outcomes (the CI bounds would otherwise not be between −1 and +1) [35, 40].

All statistical analysis was performed in Stata Version 17.0 (StataCorp, College Station, TX, USA) [41]. *P*-values were evaluated at the 0.05 level of significance.

## Results

### Descriptive statistics and weighted prevalence of child malnutrition

Table 1 presents weighted comparisons of the study variables by survey year. The sample size for the 2014 KDHS was $n = 18702$ (53%) and was $n = 16883$ (47%) for the 2022 KDHS. Between 2014 and 2022, there were significant differences in child characteristics, including age, birth interval, birth order, and delivery place; household characteristics, including religion, socioeconomic status, and region; maternal characteristics, including the mother’s age, employment status, and the father’s education ($p < 0.05$). Our analyses did not reveal substantial differences by sex and place of residence of the child during the same period ($p> 0.05$).

**Table 1:** Weighted comparisons of the study variables by survey year, KDHS 2014 and 2022

		2014	2022	
Variable	Total (*N*)	*n*(%)	*n*(%)	*p-value*
Child age, mean (SE)		29.1 (0.1)	28.5 (0.2)	0.009
Birth interval, mean (SE)		44.8 (0.4)	51.1 (0.5)	<0.001
Birth order				<0.001
1st	9919	4873 (26.1)	5046 (29.9)	
2nd	8248	4239 (22.7)	4008 (23.7)	
3rd	6099	3120 (16.7)	2979 (17.6)	
4th/5th	6672	3649 (19.5)	3023 (17.9)	
6th/higher	4647	2820 (15.1)	1827 (10.8)	
Child sex				0.765
Male	18066	9477 (50.7)	8589 (50.9)	
Female	17519	9225 (49.3)	8294 (49.1)	
Delivery place				<0.001
Home	8170	6991 (37.4)	1179 (7.0)	
Public	15160	8609 (46.0)	6551 (38.8)	
Private	5200	2855 (15.3)	2345 (13.9)	
Other	240	189 (1.0)	51 (0.3)	
Unknown/Missing	6815	57 (0.3)	6758 (40.0)	
Residence				0.339
Urban	12993	6677 (35.7)	6316 (37.4)	
Rural	22592	12025 (64.3)	10567 (62.6)	
Religion				<0.001
Catholic	6341	3370 (18.0)	2971 (17.6)	
Protestant	24749	13190 (70.5)	11559 (68.5)	
Muslim	3216	1586 (8.5)	1630 (9.7)	
Atheist	720	480 (2.6)	240 (1.4)	
Other	528	46 (0.2)	482 (2.9)	
Unknown/Missing	30	30 (0.15)	0 (0.0)	
Economic status				0.036
Poorest	8241	4457 (23.8)	3784 (22.4)	
Poorer	6841	3803 (20.3)	3038 (18.0)	
Middle	6330	3375 (18.0)	2955 (17.5)	
Richer	6695	3285 (17.6)	3410 (20.2)	
Richest	7479	3782 (20.2)	3697 (21.9)	
Mothers education				
None	3956	2218 (11.9)	1738 (10.3)	
Primary	16841	10467 (56.0)	6374 (37.8)	
Higher	14788	6016 (32.2)	8772 (52.0)	
Mothers age				0.006
under 24	10261	5575 (29.8)	4686 (27.8)	
25 - 34	18096	9492 (50.8)	8604 (50.9)	
35+	7228	3635 (19.4)	3593 (21.3)	
Mother employed				<0.001
No	11458	3240 (17.3)	8218 (48.7)	
Yes	14358	5693 (30.4)	8665 (51.3)	
Unknown/Missing	9769	9769 (52.2)	0 (0.0)	
Fathers education				<0.001
None	2142	778 (4.2)	1364 (8.1)	
Primary	9055	4178 (22.3)	4877 (28.9)	
Higher	10390	3252 (17.4)	7138 (42.3)	
Unknown/Missing	13998	10494 (56.1)	3504 (20.8)	
Region				0.033
Coast	3476	1936 (10.4)	1540 (9.1)	
N. Eastern	1247	625 (3.3)	622 (3.7)	
Eastern	4212	2235 (12.0)	1977 (11.7)	
Central	3712	1725 (9.2)	1987 (11.8)	
R. Valley	10592	5457 (29.2)	5135 (30.4)	
Western	3812	2166 (11.6)	1646 (9.7)	
Nyanza	4632	2638 (14.1)	1994 (11.8)	
Nairobi	3902	1920 (10.3)	1982 (11.74)	

Percentages may not equal exactly 100 due to rounding

Table 2 summarizes the weighted prevalence of under-five child malnutrition by selected child, household, maternal, and paternal characteristics grouped by the survey year (2014 - 2022). In 2014, 26% ($n = 4466$) of children were stunted, 11% (*n* = 1841) were underweight, and 4% (*n* = 701) were wasted. In contrast, the percentage of stunted children decreased to 18% (*n* = 2665) in 2022, while underweight and wasting decreased to 10% (*n* = 1543) and 5% (*n* = 752), respectively. This is an increase from 4% for wasting. The analyzed sample consisted of 51% male and 49% female children.

In 2014, the prevalence of stunting, underweight, and wasting was significantly higher among male children, those delivered at home, children from rural areas, and those from households with the lowest socioeconomic status. Additionally, a higher prevalence was observed among children from households identifying as Protestant $(p < 0.05)$. Most of the stunted children that year were born to mothers aged between 25 and 34 who had, at most, a primary school education $(p < 0.05)$. However, we found no significant association between maternal age, employment status, and child stunting $(p> 0.05)$. In 2022, the prevalence of stunting remained consistently higher for children with the aforementioned characteristics, except for a notably increased prevalence among children born in public hospitals.

**Table 2:** Weighted prevalence of stunting, underweight, and wasting among children under five years by selected child, household, maternal, and paternal characteristics (KDHS 2014 and 2022)

	2014						2022					
	Stunted (HAZ<−2SD)		Underweight (WAZ<−2SD)		Wasted (WHZ<−2SD)		Stunted (HAZ<−2SD)		Underweight (WAZ<−2SD)		Wasted (WHZ<−2SD)	
	*n*(%)	*p-value*	*n*(%)	*p-value*	*n*(%)	*p-value*	*n*(%)	*p-value*	*n*(%)	*p-value*	*n*(%)	*p-value*
*N*	4466 (25.8)		1841 (10.6)		701 (4.1)		2665 (17.5)		1543 (10.0)		752 (4.9)	
Child age, mean (SE)	30.8 (0.2)	<0.001	31.8 (0.5)	<0.001	26.1 (1.0)	<0.001	27.7 (0.4)	0.080	30.8 (0.5)	<0.001	30.8 (0.7)	<0.001
Birth interval, mean (SE)	39.5 (0.5)	<0.001	38.2 (0.7)	<0.001	39.8 (1.2)	<0.001	43.8 (0.8)	<0.001	41.8 (1.0)	<0.001	43.1 (1.5)	<0.001
Birth order		<0.001		<0.001		<0.001		<0.001		<0.001		<0.001
1st	910 (20.4)		342 (18.6)		149 (21.3)		651 (24.4)		330 (21.4)		158 (21.1)	
2nd	892 (20.0)		334 (18.2)		133 (19.0)		572 (21.5)		297 (19.2)		149 (19.9)	
3rd	750 (16.8)		300 (16.3)		110 (15.7)		464 (17.4)		286 (18.5)		130 (17.3)	
4th/5th	1062 (23.8)		457 (24.8)		169 (24.1)		545 (20.5)		358 (23.2)		169 (22.5)	
6th/higher	848 (19.0)		406 (22.1)		138 (19.8)		432 (16.2)		271 (17.6)		144 (19.3)	
Child sex		<0.001		<0.001		0.090		<0.001		<0.001		0.010
Male	2586 (57.9)		1028 (55.9)		382 (54.6)		1523 (57.2)		857 (55.6)		420 (55.9)	
Female	1880 (42.1)		812 (44.1)		318 (45.4)		1142 (42.8)		685 (44.4)		331 (44.1)	
Delivery place		<0.001		<0.001		<0.001		<0.001		<0.001		<0.001
Home	2157 (48.5)		1033 (56.4)		390 (55.9)		302 (16.9)		205 (23.4)		104 (25.7)	
Public	1749 (39.3)		629 (34.3)		235 (33.6)		1147 (64.2)		513 (58.6)		217 (53.1)	
Private	495 (11.1)		153 (8.4)		68 (9.7)		321 (18.0)		150 (17.2)		80 (53.1)	
Other	50 (1.1)		16 (0.9)		5 (0.7)		14 (0.8)		7 (0.8)		5 (1.4)	
Residence		<0.001		<0.001		0.030		<0.001		<0.001		0.010
Urban	1168 (26.1)		397 (21.6)		200 (28.6)		662 (24.8)		361 (23.4)		215 (28.7)	
Rural	3298 (73.9)		1443 (78.4)		500 (71.4)		2003 (75.2)		1182 (76.6)		536 (71.3)	
Religion		<0.001		<0.001		<0.001		0.010		0.03		<0.001
Catholic	728 (16.3)		296 (16.1)		126 (18.1)		445 (16.7)		276 (17.9)		128 (17.1)	
Protestant	3200 (71.8)		1257 (68.4)		444 (63.5)		1864 (70.0)		1012 (65.6)		432 (57.5)	
Muslim	346 (7.8)		195 (10.6)		106 (15.2)		232 (8.7)		190 (12.3)		159 (21.2)	
Atheist	181 (4.1)		79 (4.3)		18 (2.6)		59 (2.2)		24 (1.6)		12 (1.7)	
Other	5.4 (0.1)		8.5 (0.5)		4 (0.7)		63 (2.4)		40 (2.6)		19 (2.5)	
Economic status		<0.001		<0.001		<0.001		<0.001		<0.001		<0.001
Poorest	1489 (33.4)		792 (43.0)		303 (43.2)		986 (37.0)		679 (44.0)		340 (45.2)	
Poorer	1099 (24.6)		435 (23.7)		116 (16.6)		598 (22.4)		286 (18.5)		88 (11.8)	
Middle	808 (18.1)		286 (15.6)		117 (16.8)		439 (16.5)		249 (16.1)		116 (15.5)	
Richer	620 (13.9)		204 (11.1)		76 (11.0)		354 (13.3)		190 (12.4)		124 (16.5)	
Richest	446 (10.0)		122 (6.7)		86 (12.4)		286 (10.7)		137 (8.9)		82 (10.9)	
Mothers education		<0.001		<0.001		<0.001		<0.001		<0.001		<0.001
None	628 (14.1)		421 (22.9)		210 (30.0)		354 (13.3)		355 (23.0)		248 (33.1)	
Primary	2890 (64.7)		1118 (60.4)		329 (47.0)		1283 (48.2)		688 (44.6)		257 (34.2)	
Higher	946 (21.2)		307 (16.7)		162 (23.1)		1027 (38.6)		500 (32.4)		246 (32.7)	
Mothers age		0.200		0.010		0.350		<0.001		0.540		0.110
Under 24	1349 (30.2)		474 (25.8)		182 (26.1)		817 (30.7)		390 (25.3)		173 (23.1)	
25 - 34	2228 (49.9)		946 (51.4)		375 (53.6)		1313 (49.3)		800 (51.8)		394 (52.4)	
35+	888 (19.9)		420 (22.8)		142 (20.3)		534 (20.1)		353 (22.9)		184 (24.5)	
Mother employed		0.540		0.320		<0.001		0.200		<0.001		<0.001
No	770 (35.9)		342 (38.6)		149 (49.0)		1362 (51.1)		853 (55.3)		434 (57.8)	
Yes	1378 (64.1)		545 (61.4)		155 (51.0)		1302 (48.9)		689 (44.7)		317 (42.2)	
Fathers education		<0.001		<0.001		<0.001		<0.001		<0.001		<0.001
None	213 (10.7)		155 (18.5)		81 (27.6)		304 (14.5)		292 (23.8)		188 (30.4)	
Primary	1183 (59.3)		453 (53.9)		120 (40.8)		961 (45.8)		517 (42.0)		217 (35.1)	
Higher	599 (30.0)		232 (27.6)		93 (31.6)		833 (39.7)		419 (34.1)		213 (34.5)	
Region		<0.001		<0.001		<0.001		<0.001		<0.001		<0.001
Coast	532 (11.9)		226 (12.3)		75 (10.8)		338 (12.7)		204 (13.2)		91 (12.2)	
N. Eastern	134 (3.0)		100 (5.5)		72 (10.3)		80 (3.0)		104 (6.8)		99 (13.2)	
Eastern	640 (14.3)		259 (14.1)		97 (13.9)		377 (14.2)		206 (13.4)		108 (14.4)	
Central	289 (6.5)		78 (4.3)		33 (4.7)		232 (8.7)		94 (6.1)		46 (6.2)	
R. Valley	1502 (33.7)		772 (41.9)		289 (41.3)		950 (35.7)		623 (40.4)		287 (38.2)	
Western	506 (11.3)		164 (8.9)		41 (6.0)		231 (8.7)		117 (7.6)		31 (4.1)	
Nyanza	556 (12.5)		183 (10.0)		47 (6.8)		263 (9.9)		106 (6.9)		44 (5.9)	
Nairobi	302 (6.8)		56 (3.0)		43 (6.1)		191 (7.2)		86 (5.6)		43 (5.8)	

*HAZ* Height-for-age z-score, *WAZ *Weight-for-age z-score, *WHZ *Weight-for-height z-score, *SD* Standard deviation, *p*-value based on a Pearson chi-square test for categorical variables and t-test for continuous variables

### Changes in child malnutrition and socioeconomic inequality

Table 3 summarizes the prevalence of child malnutrition by household socioeconomic status between 2014 and 2022. Stunting and underweight decreased during this period. The absolute reduction was 9.1% and 0.6% for stunting and underweight, respectively. On the other hand, wasting prevalence was higher in 2022 compared to 2014. While the prevalence of underweight declined in 2022, this difference was not statistically significant ($p> 0.05$). A more detailed examination revealed a statistically significant decline in underweight only in the poorer socioeconomic quintile compared to stunting, where statistically significant reductions occurred across all the socioeconomic status groups.

**Table 3:** Malnutrition prevalence (%) by household socioeconomic status (KDHS 2014 and 2022)

	Poorest	Poorer	Middle	Richer	Richest	All
**Stunting (height for age < −2 SD)**						
2014	34.2 (0.6)	30.2 (0.7)	24.9 (0.8)	20.6 (0.7)	12.9 (0.7)	27.1 (0.3)
2022	25.6 (0.6)	20.5 (0.7)	15.4 (0.7)	11.7 (0.6)	07.7 (0.6)	18.0 (0.3)
Diff-1	8.6 (0.8)*	9.8 (1.0)*	9.4 (1.0)*	8.9 (1.0)*	5.2 (0.9)*	9.1 (0.4)*
**Underweight (weight for age < −2 SD)**						
2014	21.2 (0.5)	12.7 (0.5)	09.3 (0.5)	07.4 (0.5)	04.1 (0.4)	13.2 (0.2)
2022	21.8 (0.5)	10.6 (0.5)	09.6 (0.5)	06.2 (0.4)	04.5 (0.4)	12.6 (0.3)
Diff-2	−0.6 (0.7)	2.0 (0.8)*	−0.3 (0.7)	1.2 (0.7)	−0.3 (0.6)	0.6 (0.3)
**Wasting (weight for height < −2 SD)**						
2014	09.4 (0.4)	03.6 (0.3)	03.8 (0.3)	03.2 (0.3)	02.9 (0.3)	05.5 (0.2)
2022	12.9 (0.4)	04.2 (0.4)	05.3 (0.4)	04.3 (0.4)	02.9 (0.3)	07.2 (0.2)
Diff-3	−3.5 (0.6)*	−0.6 (0.4)	−1.6 (5.3)*	−1.1 (0.5)	0.0 (0.5)	−1.7 (0.3)*

Diff-1, Diff-2, Diff-3: difference in under five stunting, underweight, and wasting, respectively; SE: standard error; SD: standard deviation

^*^$$p < 0.05$$

**Fig. 1:**
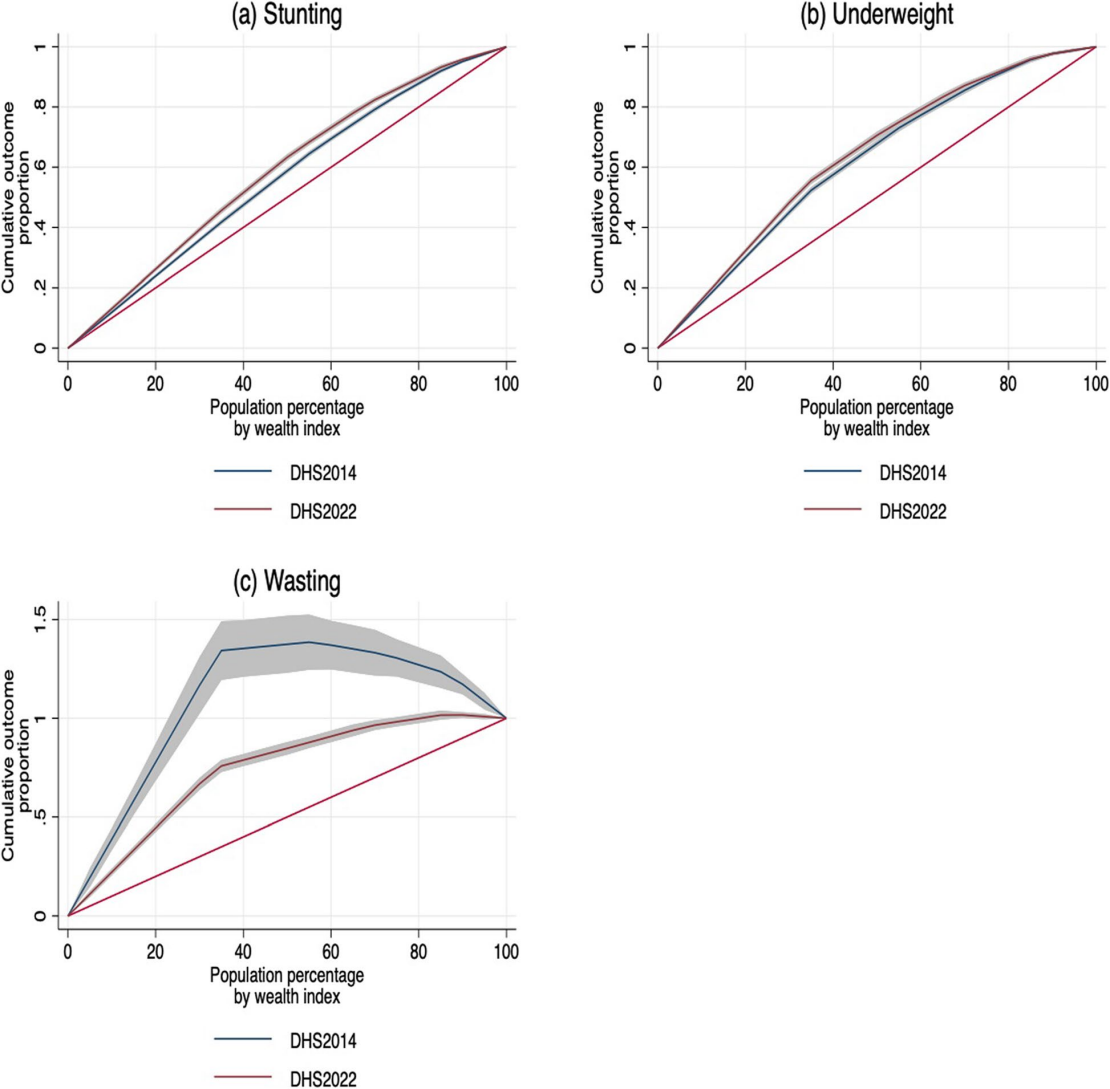
Concentration curves for stunting, underweight, and wasting in Kenya (2014 and 2022). All concentration curves are above the line of equity (red), suggesting that inequality in under-five child malnutrition is higher among the poorest socioeconomic quintiles

Table 4 presents the concentration indices of under-five child malnutrition. The CIs of stunting and underweight were significantly different from 0 between 2014 and 2022 ($p < 0.001$), whereas wasting did not show a significant difference during this period ($p> 0.05$). All differences in CIs were negative, suggesting that children from the poorest socioeconomic groups are more likely to be stunted, underweight, or wasted relative to those from the richest households. Additionally, absolute values of the CIs of stunting and underweight in 2022 were higher than in 2014, suggesting that inequalities in under-five child underweight and stunting increased during this period (Fig. 1a - b). On the other hand, the CI for wasting in 2022 was lower relative to 2014, suggesting that the inequality in child wasting declined during this period (Fig. 1c). The difference in the CI for wasting between 2014 and 2022 was, however, not significant ($p> 0.05$).

**Table 4:** Under-five child malnutrition concentration indices (CI) (KDHS 2014 and 2022)

	Stunted (HAZ < −2 SD)		Underweight (WAZ < −2 SD)		Wasted (WHZ < −2 SD)	
	CI (SE)	p-value*	CI (SE)	p-value*	CI (SE)	p-value*
Year 2014	−0.15 (0.01)	<0.001	−0.27 (0.02)	<0.001	12.37 (22.61)	0.580
Year 2022	−0.79 (0.01)	<0.001	−0.88 (0.01)	<0.001	−1.96 (0.05)	<0.001
Diff	−0.64 (0.01)	<0.001	−0.61 (0.02)	<0.001	−14.33 (22.62)	0.530

*Diff *Difference in child malnutrition concentration indices between 2014 and 2022, *SE *Standard error, *SD *Standard deviation, *HAZ* Height-for-age Z-score, *WAZ* Weight-for-age Z-score, *WHZ* Weight-for-height Z-score

^*^ *p*-value based on a two-tailed independence test

### Determinants of under-five child malnutrition

Table 5 summarizes the determinants of under-five child stunting, underweight, and wasting based on unadjusted logistic regression. Results are based on analyzing the aggregate 2014 and 2022 KDHS datasets. We found evidence indicating that male children have an increased risk of all three indicators of malnutrition. Factors such as the child’s increasing age, being a thirdborn or higher, household socioeconomic status (with poorer, middle, and richer households at increased risk compared to the richest), and region (including Coast, Northeastern, Eastern, Rift Valley, Western, and Nyanza compared to Nairobi) were significantly associated with an increased risk of stunting and underweight. Additionally, mothers aged 35 years and older were more likely to have children who were underweight or wasted. Furthermore, having a birth order of fourth or higher and being of the Muslim faith were linked to an increased risk of wasting.

**Table 5:** Unadjusted analysis of factors associated with under-five child malnutrition (KDHS 2014 and 2022)

	Stunted (HAZ<−2SD)		Underweight (WAZ<−2SD)		Wasted (WHZ<−2SD)	
	OR(95%CI)	*p-value*	OR(95%CI)	*p-value*	OR(95%CI)	*p-value*
Year						
KDHS 2014	ref		ref		ref	
KDHS 2022	0.60 (0.56 - 0.66)	<0.001	0.94 (0.84 - 1.05)	0.244	1.22 (1.05 - 1.43)	0.011
Child age	1.00 (1.00 - 1.01)	<0.001	1.01 (1.01 - 1.01)	<0.001	1.00 (1.00 - 1.00)	0.797
Birth interval	0.99 (0.99 - 0.99)	<0.001	0.99 (0.99 - 0.99)	<0.001	0.99 (0.99 - 0.99)	<0.001
Birth order						
1st	ref		ref		ref	
2nd	1.08 (0.98 - 1.19)	0.130	1.07 (0.93 - 1.23)	0.323	1.04 (0.85 - 1.27)	0.687
3rd	1.22 (1.09 - 1.36)	<0.001	1.35 (1.16 - 1.56)	<0.001	1.18 (0.95 - 1.47)	0.129
4th/5th	1.53 (1.39 - 1.69)	<0.001	1.74 (1.53 - 1.99)	<0.001	1.52 (1.25 - 1.84)	<0.001
6th/higher	1.83 (1.63 - 2.04)	<0.001	2.12 (1.86 - 2.43)	<0.001	1.83 (1.52 - 2.21)	<0.001
Child sex						
Female	ref		ref		ref	
Male	1.43 (1.35 - 1.53)	<0.001	1.26 (1.16 - 1.37)	<0.001	1.21 (1.07 - 1.38)	0.003
Residence						
Rural	ref		ref		ref	
Urban	0.58 (0.52 - 0.64)	<0.001	0.51 (0.45 - 0.58)	<0.001	0.74 (0.63 - 0.88)	0.001
Religion						
Atheist	ref		ref		ref	
Catholic	0.46 (0.36 - 0.58)	<0.001	0.60 (0.47 - 0.78)	<0.001	0.96 (0.63 - 1.46)	0.844
Protestant	0.51 (0.41 - 0.64)	<0.001	0.61 (0.48 - 0.77)	<0.001	0.83 (0.56 - 1.24)	0.357
Muslim	0.44 (0.35 - 0.56)	<0.001	0.83 (0.64 - 1.08)	0.164	2.05 (1.35 - 3.13)	0.001
Other	0.30 (0.20 - 0.45)	<0.001	0.61 (0.41 - 0.93)	0.020	1.06 (0.57 - 1.97)	0.845
Wealth index						
Poorest	3.68 (3.15 - 4.29)	<0.001	5.59 (4.48 - 6.98)	<0.001	3.37 (2.60 - 4.38)	<0.001
Poorer	2.79 (2.38 - 3.27)	<0.001	3.00 (2.39 - 3.77)	<0.001	1.22 (0.92 - 1.63)	0.169
Middle	2.11 (1.79 - 2.49)	<0.001	2.39 (1.88 - 3.05)	<0.001	1.55 (1.16 - 2.08)	0.003
Richer	1.52 (1.27 - 1.81)	<0.001	1.68 (1.31 - 2.15)	<0.001	1.29 (0.96 - 1.73)	0.087
Richest	ref		ref		ref	
Mothers education						
None	ref		ref		ref	
Primary	1.00 (0.90 - 1.11)	0.975	0.49 (0.44 - 0.54)	<0.001	0.27 (0.24 - 0.32)	<0.001
Higher	0.47 (0.42 - 0.53)	<0.001	0.24 (0.21 - 0.27)	<0.001	0.22 (0.18 - 0.26)	<0.001
Mothers age						
under 24	ref		ref		ref	
25 - 34	0.86 (0.79 - 0.94)	<0.001	1.11 (1.00 - 1.23)	0.054	1.18 (1.01 - 1.38)	0.033
35+	0.84 (0.76 - 0.93)	<0.001	1.22 (1.07 - 1.38)	0.003	1.23 (1.03 - 1.47)	0.021
Mother employed						
No	ref		ref		ref	
Yes	1.04 (0.95 - 1.13)	0.455	0.83 (0.74 - 0.93)	0.001	0.65 (0.55 - 0.76)	<0.001
Fathers education						
None	ref		ref		ref	
Primary	0.94 (0.84 - 1.06)	0.319	0.44 (0.38 - 0.50)	<0.001	0.26 (0.21 - 0.31)	<0.001
Higher	0.49 (0.43 - 0.56)	<0.001	0.25 (0.21 - 0.29)	<0.001	0.21 (0.17 - 0.26)	<0.001
Delivery place						
Home	ref		ref		ref	
Public sector	0.55 (0.50 - 0.60)	<0.001	0.46 (0.41 - 0.51)	<0.001	0.48 (0.41 - 0.56)	<0.001
Private sector	0.42 (0.37 - 0.48)	<0.001	0.34 (0.29 - 0.41)	<0.001	0.46 (0.36 - 0.59)	<0.001
Other	0.83 (0.58 - 1.20)	0.325	0.59 (0.37 - 0.96)	0.033	0.72 (0.33 - 1.55)	0.396
Region						
Nairobi	ref		ref		ref	
Coast	2.11 (1.63 - 2.74)	<0.001	3.41 (2.28 - 5.09)	<0.001	2.04 (1.25 - 3.31)	0.004
N. Eastern	1.35 (1.01 - 1.80)	0.041	4.94 (3.31 - 7.37)	<0.001	6.69 (4.21 - 10.63)	<0.001
Eastern	1.93 (1.50 - 2.49)	<0.001	2.90 (1.93 - 4.35)	<0.001	2.01 (1.25 - 3.23)	0.004
Central	1.05 (0.79 - 1.39)	0.730	1.20 (0.76 - 1.89)	0.431	0.90 (0.52 - 1.56)	0.713
R. Valley	1.89 (1.48 - 2.41)	<0.001	3.65 (2.48 - 5.38)	<0.001	2.31 (1.45 - 3.66)	<0.001
Western	1.49 (1.15 - 1.94)	0.003	1.90 (1.26 - 2.88)	0.002	0.78 (0.46 - 1.31)	0.344
Nyanza	1.32 (1.03 - 1.70)	0.028	1.58 (1.06 - 2.37)	0.025	0.81 (0.49 - 1.33)	0.396

*OR* Odds Ratio, *CI* Confidence Interval, Wealth index is used as a proxy for a household’s socioeconomic status

Table 6 summarizes determinants of under-five child malnutrition based on adjusted multivariable logistic regression. Birth order was excluded from the adjusted model due to multicollinearity. We found that a child’s age (in months) (AOR = 1.01; 95% Confidence Interval [CI]: 1.01 – 1.02) and their sex (male) (AOR = 1.50; 95%CI: 1.35 – 1.67) were significantly associated with an increased risk of stunting. In addition, the odds of stunting were higher for children from households in the poorest (AOR = 2.67; 95%CI: 1.92 – 3.72), poorer (AOR = 2.06; 95%CI: 1.50 – 2.84), middle (AOR = 1.78; 95%CI: 1.29 - 2.45), and richer (AOR = 1.53; 95%CI: 1.11 - 2.10) socioeconomic quintiles relative to those from households in the wealthiest socioeconomic status group.

**Table 6:** Adjusted analyses of determinants of under-five child malnutrition (KDHS 2014 and 2022)

	Stunted (HAZ<−2SD)		Underweight (WAZ<−2SD)		Wasted (WHZ<−2SD)	
	AOR 95%CI	*p-value*	AOR 95%CI	*p-value*	AOR 95%CI	*p-value*
Year						
KDHS 2014	ref		ref		ref	
KDHS 2022	0.89 (0.78 - 1.03)	0.120	1.20 (1.01 - 1.42)	0.039	1.25 (0.98 - 1.58)	0.073
Child age	1.01 (1.01 - 1.02)	<0.001	1.01 (1.01 - 1.02)	<0.001	0.99 (0.98 - 1.00)	0.011
Birth interval	0.99 (0.99 - 1.00)	<0.001	0.99 (0.99 - 1.00)	<0.001	0.99 (0.99 - 1.00)	0.029
Child sex						
Female	ref		ref		ref	
Male	1.50 (1.35 - 1.67)	<0.001	1.43 (1.24 - 1.65)	<0.001	1.29 (1.05 - 1.59)	0.015
Residence						
Rural	ref		ref		ref	
Urban	1.06 (0.90 - 1.25)	0.463	1.14 (0.93 - 1.41)	0.205	1.43 (1.08 - 1.89)	0.012
Religion						
Atheist	ref		ref		ref	
Catholic	0.77 (0.54 - 1.10)	0.153	1.29 (0.82 - 2.03)	0.262	0.96 (0.50 - 1.85)	0.910
Protestant	0.85 (0.61 - 1.19)	0.352	1.25 (0.82 - 1.91)	0.309	1.16 (0.63 - 2.13)	0.624
Muslim	0.67 (0.45 - 1.02)	0.060	0.84 (0.49 - 1.41)	0.500	1.03 (0.50 - 2.13)	0.935
Other	0.88 (0.49 - 1.59)	0.672	1.52 (0.76 - 3.03)	0.233	1.41 (0.55 - 3.58)	0.475
Wealth index						
Poorest	2.67 (1.92 - 3.72)	<0.001	2.20 (1.45 - 3.35)	<0.001	1.82 (1.05 - 3.14)	0.032
Poorer	2.06 (1.50 - 2.84)	<0.001	1.36 (0.91 - 2.04)	0.138	0.83 (0.48 - 1.44)	0.499
Middle	1.78 (1.29 - 2.45)	<0.001	1.33 (0.89 - 2.00)	0.159	1.56 (0.92 - 2.65)	0.096
Richer	1.53 (1.11 - 2.10)	0.008	1.07 (0.71 - 1.60)	0.758	1.13 (0.69 - 1.84)	0.627
Richest	ref		ref		ref	
Mothers education						
None	ref		ref		ref	
Primary	1.20 (0.98 - 1.47)	0.076	0.93 (0.74 - 1.17)	0.518	0.69 (0.51 - 0.95)	0.021
Higher	0.88 (0.68 - 1.12)	0.294	0.59 (0.43 - 0.80)	0.001	0.55 (0.35 - 0.85)	0.627
Mothers age						
under 24	ref		ref		ref	
25 - 34	0.95 (0.80 - 1.12)	0.511	1.54 (1.24 - 1.92)	<0.001	1.38 (1.04 - 1.83)	0.026
35+	0.89 (0.73 - 1.07)	0.208	1.53 (1.19 - 1.96)	0.001	1.50 (1.09 - 2.07)	0.013
Mother employed						
No	ref		ref		ref	
Yes	1.08 (0.95 - 1.23)	0.247	1.04 (0.88 - 1.23)	0.648	0.93 (0.73 - 1.18)	0.561
Fathers education						
None	ref		ref		ref	
Primary	1.06 (0.87 - 1.30)	0.553	0.80 (0.63 - 1.02)	0.071	0.74 (0.53 - 1.04)	0.086
Higher	0.89 (0.70 - 1.13)	0.326	0.83 (0.62 - 1.10)	0.193	0.80 (0.52 - 1.21)	0.286
Delivery place						
Home	ref		ref		ref	
Public hosp.	0.85 (0.73 - 0.99)	0.032	0.75 (0.63 - 0.90)	0.002	0.80 (0.60 - 1.06)	0.125
Private hosp.	0.95 (0.76 - 1.18)	0.637	0.85 (0.64 - 1.12)	0.244	0.96 (0.64 - 1.44)	0.832
Other	1.25 (0.75 - 2.07)	0.388	1.29 (0.68 - 2.48)	0.436	2.03 (0.81 - 5.07)	0.131
Region						
Nairobi	ref		ref		ref	
Coast	0.80 (0.51 - 1.26)	0.343	1.52 (0.86 - 2.71)	0.152	1.13 (0.52 - 2.44)	0.756
N. Eastern	0.48 (0.29 - 0.78)	0.003	1.54 (0.87 - 2.73)	0.141	2.09 (0.98 - 4.47)	0.056
Eastern	0.76 (0.50 - 1.16)	0.205	1.46 (0.85 - 2.51)	0.172	1.54 (0.74 - 3.19)	0.246
Central	0.58 (0.36 - 0.94)	0.027	0.98 (0.53 - 1.83)	0.959	0.76 (0.32 - 1.81)	0.533
R. Valley	0.67 (0.44 - 1.02)	0.061	1.44 (0.84 - 2.46)	0.18	1.41 (0.68 - 2.89)	0.353
Western	0.49 (0.31 - 0.76)	0.002	0.78 (0.43 - 1.41)	0.417	0.63 (0.28 - 1.44)	0.271
Nyanza	0.44 (0.29 - 0.68)	<0.001	0.80 (0.46 - 1.41)	0.445	0.64 (0.30 - 1.39)	0.261

*AOR* Adjusted Odds Ratio, *CI *Confidence Interval, Wealth index is a proxy for socioeconomic status

We found similarly that a child’s age (AOR = 1.01; 95%CI: 1.01 - 1.02), sex (male) (AOR = 1.43; 1.24 - 1.65), and being born to a household in the poorest socioeconomic quintile (AOR = 2.20; 95%CI: 1.45 - 3.35) were associated with an increased risk of underweight. Furthermore, children born to mothers between 25 and 34 years (AOR = 1.54; 95%CI: 1.24 - 1.92) and those above 35 years (AOR = 1.53; 95%CI: 1.19 - 1.96) were at an increased risk of underweight compared to those born to women under 25 years.

Factors associated with an increased risk of wasting included the child’s sex (male) (AOR = 1.29; 95%CI: 1.05 – 1.59), urban residence (AOR = 1.43; 95%CI: 1.08 – 1.89), being born to a house in the poorest socioeconomic quintile (AOR = 1.82; 95%CI: 1.05 – 3.14), being born to a mother aged between 26 and 34 years (AOR = 1.38; 95%CI: 1.04 – 1.83) or being born to a mother aged 35 years and above (AOR = 1.50; 95%CI: 1.09 – 2.07).

### Under-five child stunting screening

Table 7 summarizes the results based on screening for child stunting using a household’s socioeconomic status, child’s age, and sex, respectively. The sensitivity of a household’s socioeconomic status as a screening tool for under-five child malnutrition was 67.4% (95% CI: 66.4% - 68.4%). The specificity was 50.6% (95% CI: 50.0% - 51.1%). The ability of a household’s socioeconomic status to discriminate between stunted and non-stunted children was above random guessing (AUC = 0.59; 95% CI: 0.58 - 0.60).

**Table 7:** Screening for child stunting using a household’s socioeconomic status (SES), child’s age, sex, and birth order number

	Metric	95% CI
**Socioeconomic status**		
	Sensitivity	67.4 (66.4 - 68.4)
	Specificity	50.6 (50.0 - 51.1)
	AUC	0.59 (0.58 - 0.60)
	NPV	84.1 (83.5 - 84.6)
	PPV	28.6 (28.0 - 29.2)
**Child’s age**		
	Sensitivity	49.6 (48.5 - 50.7)
	Specificity	52.6 (52.0 - 53.2)
	AUC	0.51 (0.50 - 0.52)
	NPV	78.0 (77.4 - 78.6)
	PPV	23.5 (22.9 - 24.2)
**Child’s sex**		
	Sensitivity	57.0 (55.9 - 58.0)
	Specificity	51.2 (50.6 - 51.8)
	AUC	0.54 (0.53 - 0.55)
	NPV	80.2 (79.6 - 80.8)
	PPV	25.5 (24.9 - 26.2)

SES: Socioeconomic status; AUC: Area under the curve; NPV: Negative predictive value; PPV: Positive predictive value

On the other hand, the sensitivity, specificity, and AUC values based on screening using the child’s age were 49.6% (95% CI: 48.5% - 50.7%), 52.6% (95% CI: 52.0% - 53.2%), and 0.51 (95% CI: 0.50 - 0.52), respectively. The sensitivity and specificity values based on screening for under-five child stunting using the child’s sex were 57.0% (95% CI: 55.9% - 58.0%) and 51.2% (95% CI: 50.6% - 51.8%), respectively. The discriminatory ability based on using the child’s age was limited (AUC = 0.51; 95% CI: 0.50 - 0.52), whereas that based on using sex (AUC = 0.54; 95% CI: 0.53 - 0.55) was slightly above average.

### Decomposition of the concentration indices for stunting and underweight

In Table 8, we present each determinant of child malnutrition and its contribution to the observed inequality in child stunting and underweight for 2014 and 2022. We decomposed the CIs of stunting and underweight, child malnutrition indicators which differed significantly between 2014 and 2022. A household’s socioeconomic status (1.003), maternal education (0.298), and birth interval (0.108) contributed the most toward the observed inequality in child stunting in 2014. The contribution of a household’s socioeconomic status increased to 1.8 in 2022, whereas that of maternal education decreased to 0.001. The contribution of paternal education to inequality in child stunting became more pronounced in 2022 relative to 2014 (0.292). On the other hand, the contribution of a household’s socioeconomic status to inequality in under-five child underweight declined in 2022 to 0.936 from 1.371 in 2014. Even so, a household’s socioeconomic status remained the chief contributor to inequality in both years. The contribution of maternal education was 0.440 in 2014 and increased to 0.556 in 2022.

**Table 8:** Decomposition of the concentration indices and contributions of determinants of under-five child stunting and underweight, 2014 and 2022

	Stunting				Underweight			
	2014		2022		2014		2022	
	CI	Contribution	CI	Contribution	CI	Contribution	CI	Contribution
Child sex	−0.002	−0.003	−0.003	−0.005	−0.002	−0.002	−0.003	−0.004
Residence	−0.540	−0.267	−0.617	−0.643	−0.540	−0.324	−0.617	−0.631
Religion	−0.050	−0.002	−0.019	−0.001	−0.050	−0.019	−0.020	−0.018
Mothers education	0.390	0.298	0.449	−0.097	0.390	0.440	0.449	0.556
Mothers age (years)	−0.012	−0.004	0.040	0.001	−0.012	0.008	0.040	−0.006
Mother employed	0.078	−0.024	0.180	−0.021	0.078	−0.011	0.180	0.005
Fathers education	0.404	−0.001	0.494	0.292	0.404	0.006	0.494	0.381
Delivery place	0.332	0.064	0.181	−0.048	0.332	0.105	0.181	0.003
Region	0.131	0.013	0.122	0.082	−0.048	0.151	0.122	0.087
Birth interval (months)	0.110	0.108	0.098	0.077	0.110	0.100	0.098	0.063
Child age (months)	0.003	−0.003	−0.002	0.006	0.003	−0.003	−0.002	0.003
Socioeconomic status	0.677	1.003	0.693	1.809	0.677	1.371	0.693	0.936

Contribution represents how much of the overall socioeconomic-related health inequality is attributed to a specific variable. Negative values of this quantity suggest that a variable contributes to reductions in the overall socioeconomic-related inequality in child malnutrition (stunting and underweight)

## Discussion

This study aimed to investigate the trends of socioeconomic inequalities in child malnutrition in Kenya. We also sought to identify key determinants, assess the utility of these determinants in screening for chronic malnutrition, and quantify their contributions to disparities in child malnutrition. Our findings indicate a notable decrease in the prevalence of stunting and underweight between 2014 and 2022, with stunting showing the highest decline. However, despite these improvements, socioeconomic disparities in child malnutrition worsened, particularly for stunting and underweight. A household’s socioeconomic status was the most significant contributor to the overall inequality in child malnutrition and was a vital determinant of a child’s nutritional status. We also found that the contribution of a father’s education to the overall inequality in child stunting rose between 2014 and 2022. Our analysis of the utility of a household’s socioeconomic status in screening for chronic child malnutrition revealed that this indicator is better than using a child’s age and sex, and is marginally better than random guessing.

This study used data from a nationally representative population-based survey, and the findings present several crucial policy implications, laying the groundwork for addressing child malnutrition in Kenya.

First, our analysis revealed that male children consistently exhibited higher prevalence rates for all three malnutrition indicators (stunting, underweight, and wasting) compared to female children. This sex disparity aligns with previous studies suggesting that male children are more vulnerable to malnutrition due to their faster growth rates, higher biological fragility [42], and potential preferential treatment of female children [32, 43]. We also found that older children are more likely to be stunted, a finding we argue could be due to less attention and care from their parents [44] or the fact that as children grow older, their body requires more energy. For example, with births spaced quickly, high-order children are likely to receive limited lacteal feeding, a factor that likely predisposes them to malnutrition [45]. This difference in child malnutrition by the child’s sex and age alludes to the need for an educational intervention involving community-adapted strategies to educate parents on the importance of equal nutritional care for children regardless of sex or age [46].

Second, there is a need for policies focusing on inclusive economic growth that benefits all socioeconomic groups. For example, implementing progressive taxation policies can help redistribute wealth and fund social programs. Additional steps could include creating employment opportunities accessible to low-income individuals, and ensuring economic development projects include provisions for improving the living standards of the poorest households. These actions could enhance equity in the distribution of the proceeds from economic growth and reduce disparities in the child malnutrition burden between socioeconomic quintiles.

In our analyses, we found the prevalence of child malnutrition to be higher among children born to households in the poorest socioeconomic groups. The risk of child malnutrition is significantly reduced with improvements in socioeconomic status due to purchasing power, improved access to quality food, and healthcare [47, 48]. Additionally, concentration indices (in absolute values) increased between 2014 and 2022 for stunting and underweight, suggesting that socioeconomic disparities in these malnutrition indicators worsened between these years. Similarly, the contribution of the household’s socioeconomic status to inequality in child malnutrition increased between 2014 and 2022, even though the overall malnutrition prevalence decreased.

Although Kenya experienced economic growth during the study period, our findings suggest a disconnect between this growth and the distribution of wealth in the general population. This disconnect has also been highlighted elsewhere [4, 8, 11, 49]. The benefits of economic growth are concentrated among the wealthier segments of society, leaving the poorer segments with limited access to resources and opportunities. Moreover, although incentive programs have played a crucial role in lowering the prevalence of child malnutrition, the rapid population growth experienced over the years has not kept pace with the economic growth rate. Consequently, a larger portion of the population has remained in poverty [33]. The disparities witnessed in the population’s economic status have worked to reduce access to essential services for people with low incomes. This has resulted in the rich having access to high-quality education, healthcare, and food, while the poor struggle to meet their basic needs [50–52]. With limited finances, a household’s ability to afford a stable supply of quality food is significantly reduced, consequences of which include adverse effects on child growth and cognitive development [53–57]. Our findings thus bring forth critical ethical considerations for designing targeted policies and programs prioritizing nutritional improvements for children from households in the lower wealth groups.

Third, we found a consistently high prevalence of all three child malnutrition indicators in the rural areas compared to urban areas. This finding underscores the importance of rural reforms, including projects that aim to bridge the gap between rural and urban development through equitable distribution of resources. Policies should strive to improve infrastructure, healthcare services, and economic opportunities in rural regions to address the disparities in child malnutrition.

While Kenya has made commendable strides in improving nutritional outcomes through such programs as the provision of subsidized fertilizers to farmers to improve agricultural production, which is a significant source of income in rural areas, this has been challenged by procurement delays, tedious access processes, and low nutrient composition [58]. This has reduced agricultural production, contributed to food insecurity, and, ultimately, poor nutritional outcomes. Moreover, the government has sustained high spending on education, with its total expenditure reaching international benchmarks and surpassing that of other low- and middle-income countries in the region. This has resulted in admirable primary and secondary school outcomes, including increased overall enrolment and improved numeracy. Despite these advances, the country still faces substantial regional inequalities in educational outcomes. Rural areas have had significantly lower net enrollment rates, with most of this concentrated in the north and northeast regions and aggravated by low income [59]. These disparities in educational and nutritional outcomes between rural and urban areas allude to the need for rural development reforms emphasizing equity in the allocation of resources.

Fourth, the COVID-19 pandemic significantly exacerbated existing inequalities and challenged public health systems [60]. For example, studies have reported the role of government-imposed COVID-19 restrictions in disrupting the food supply chain, reducing the income available to households, and contributing to food insecurity [61, 62]. The link between COVID-19, food insecurity, and health system disturbances points to a need for robust social justice-centered pandemic preparedness measures [63]. These could include incorporating social safety nets in future pandemic preparedness measures and strengthening the healthcare system through health surveillance systems to monitor and promptly address emerging health threats [64]. By strengthening healthcare systems, Kenya can build resilience against future health crises and reduce the impact of pandemics on vulnerable populations (low-income groups).

Fifth, the observed inequalities in child malnutrition point to the need for reforms in the monitoring and evaluation (M&E) of child malnutrition rates and the effectiveness of interventions implemented to keep the malnutrition indicators in check. While the current M&E system provides a foundation, it requires enhancements to address the complexities of malnutrition and socioeconomic inequality. Policymakers should establish robust systems to track progress and make data-driven adjustments to policies and programs to ensure that interventions are effective and responsive to changing needs [65].

Our findings highlighted a dependence of malnutrition indicators on several factors, including a child’s age, sex, and socioeconomic factors, similar to those reported elsewhere [8, 9, 11]. Integrating data from multiple sectors can provide a holistic view of the determinants of malnutrition. Specific actions could include developing a centralized data repository that consolidates information from the health, agriculture, education, and social protection sectors. This will enable comprehensive analysis and informed decision-making. In addition, the observed differences in malnutrition rates between rural-urban and poor-rich households highlight the importance of community-based monitoring of the child malnutrition indicators to ensure that the data collected is accurate and interventions are sufficient. For example, while the government has implemented feeding programs to improve nutritional outcomes, there is a lack of diversity and balance in the meals provided, insufficient government funding, an overreliance on donor funding, and limited parental involvement [66]. Establishing community feedback mechanisms to ensure that local insights inform policy and program design could be critical in addressing the disparities in child malnutrition.

Lastly, in assessing the nutritional status of children, anthropometric data – weight-for-height, height-for-age, and weight-for-age – are often collected. These data are usually prone to random error due to the complexities involved in their collection in younger children [32]. We explored the clinical usefulness of a household’s socioeconomic status, a child’s sex, and age in diagnosing child stunting. A household’s socioeconomic status demonstrated some potential compared to using the child’s age, sex, and random guessing. While identifying stunting based on socioeconomic status was better than using age and sex alone and slightly more accurate than random guessing, it still suggests that this assessment method may lack sufficient accuracy. Therefore, there is a need to develop screening tools that consider multiple indicators to enhance precision. For instance, socioeconomic status is often assessed using proxies like the wealth index, which may not accurately represent a household’s economic conditions. Furthermore, malnutrition is influenced by various factors. While focusing on a single factor, like socioeconomic status, can be cost-effective, it may not provide a complete understanding of the issue. Consequently, it is crucial to integrate these insights into existing health and social programs to create practical alternative solutions for resource-limited settings.

The following limitations should be considered while interpreting the findings of this study. First, due to reliance on cross-sectional study data, our findings cannot be construed as suggesting a causal relationship between socioeconomic indices and child malnutrition. Secondly, residence was classified as either urban or rural. Classifying this variable into these two localities might pose a problem following the heterogeneity associated with large cities and the unavailability of data to quantify these dissimilarities. Future work could explore these indices by county to give more refined results at this level. Third, our analysis adjusted for potentially confounding variables that did not have substantial missingness and might not include all possible confounders. However, our employment of standard statistical practices and the included covariates allow for a robust analysis of the scope of the problem.

## Conclusion

Understanding the dynamics of under-five child malnutrition and its variations by socioeconomic quintiles is crucial in keeping the existing disparities under check. Our analyses revealed an increase in socioeconomic inequality in under-five child malnutrition between 2014 and 2022 despite an overall decrease in prevalence. We found subtle differences in child malnutrition prevalence by region of residence, alluding to rural-urban differences in resource distribution. We found a child’s age, sex, and a household’s socioeconomic status to be significant determinants of child malnutrition. Decomposition analyses revealed a household’s socioeconomic status to be the most important contributor to the total observed inequality in child stunting and underweight. This result highlights that even though there has been economic growth in the past decade, there is a disconnect between this growth and how the profits are distributed between social classes.

The implications of these findings are profound. That is, increased socioeconomic disparities exacerbate the risk of malnutrition among children from poorer households, leading to long-term adverse effects on their growth, cognitive development, and overall health. Addressing these disparities requires targeted interventions that focus on improving the socioeconomic conditions of the most vulnerable populations. Efforts should be directed towards enhancing access to quality healthcare, education, and nutrition for children from low-income households. By doing so, we can work towards reducing the gap in health outcomes and ensuring that all children have the opportunity to thrive, regardless of their socioeconomic background.

## Data Availability

Data analyzed in this study is available from the Demographic and Health Survey at https://dhsprogram.com/data/ on reasonable request.
